# Molecular Assay Development to Monitor the Kinetics of Viable Populations of Two Biocontrol Agents, *Bacillus subtilis* QST 713 and *Gliocladium catenulatum* J1446, in the Phyllosphere of Lettuce Leaves

**DOI:** 10.3390/biology10030224

**Published:** 2021-03-15

**Authors:** Gurkan Tut, Naresh Magan, Philip Brain, Xiangming Xu

**Affiliations:** 1 NIAB East Malling Research, West Malling, Kent ME19 6BJ, UK; Gurkan.Tut@hotmail.com (G.T.); philip.brain@emr.ac.uk (P.B.); Xiangming.Xu@niab.com (X.X.); 2 Applied Mycology Group, Environment and AgriFood Theme, Cranfield University, Cranfield, Bedford MK43 0AL, UK

**Keywords:** biocontrol, commercial formulations, quantitative method, qPCR, PMA, phyllosphere, viable cells

## Abstract

**Simple Summary:**

There is a need to be able to track the viable populations of biocontrol agents when applied on the foliar surfaces of plants. We have developed a molecular-based method for the quantification of viable cells of two commercial biocontrol agents—a bacterium (*Bacillus subtilis*) and a fungus (*Gliocladium catenulatum*). The method has been tested on the leaf surfaces of lettuce plants to examine the changes in viable population over 10–12 days for the first time.

**Abstract:**

Optimising the use of biocontrol agents (BCAs) requires the temporal tracking of viable populations in the crop phyllosphere to ensure that effective control can be achieved. No sensitive systems for quantifying viable populations of commercially available BCAs, such as *Bacillus subtilis* and *Gliocladium catenulatum*, in the phyllosphere of crop plants are available. The objective of this study was to develop a method to quantify viable populations of these two BCAs in the crop phyllosphere. A molecular tool based on propidium monoazide (PMA) (PMAxx™-qPCR) capable of quantifying viable populations of these two BCAs was developed. Samples were treated with PMAxx™ (12.5–100 μM), followed by 15 min incubation, exposure to a 800 W halogen light for 30 min, DNA extraction, and quantification using qPCR. This provided a platform for using the PMAxx™-qPCR technique for both BCAs to differentiate viable from dead cells. The maximum number of dead cells blocked, based on the DNA, was 3.44 log_10_ for *B. subtilis* and 5.75 log_10_ for *G. catenulatum*. Validation studies showed that this allowed accurate quantification of viable cells. This method provided effective quantification of the temporal changes in viable populations of the BCAs in commercial formulations on lettuce leaves in polytunnel and glasshouse production systems.

## 1. Introduction

The efficacy of a biocontrol agent (BCA) is usually evaluated by the dose response relationships against their target pathogens [1]. This requires accurate quantification of the viable populations in the applied formulations in relation to climatic conditions and relative control of the target pathogen. Traditionally, this is done by culturing the viable populations or microscopic counts. However, such methods are very labour-intensive [2]. In addition, some propagules or cells of bacterial BCAs may remain viable under environmental stress conditions by entering a hibernation phase, which may influence the total viable populations present [3,4].

BCA detection/quantification has been improved with nucleic acid-targeting techniques such as the polymerase chain reaction (PCR) and quantitative-PCR (qPCR) [5]. PCR lacks specificity for quantitative research as the amplification curves are absent, and results are dependent on the end product [6]. Such qPCR systems have been developed and used to detect and quantify BCA populations of *Pantoea agglomerans* [7], *Pseudomonas fluorescens* [8], and *Cryptosporiopsis kienholzii* [9]. However, the conventional qPCR technique cannot discriminate between viable (live), non-viable (dead) cells, or extracellular DNA fragments, because DNA from all these are normally quantified. This limits the understanding of the population dynamics of formulated BCAs [10].

Propidium monoazide (PMA), a membrane-impermeable nucleic acid intercalating dye, when used as a pre-treatment before qPCR, enables the blocking of DNA from dead cells, which results in the sole quantification of DNA from live cells [10,11]. PMA enters cells with ruptured membranes (i.e., dead cells) and intercalates with the cell DNA. The PMA-DNA complex, once photo-activated, becomes a permanent structure as the PMA dye releases an azido group resulting in a covalent cross-linkage between the PMA and the DNA complex. This irreversible change in DNA polymerase structure obstructs access to the modified DNA and therefore stops amplification by PCR techniques [11,12,13]. The success of the PMA-qPCR in quantifying viable propagules from fungal taxa populations has been reported for *Listeria monocytogenes*, *Salmonella* and *E. coli* species within simple matrices [14], identifying propagules of fungal taxa such as *Alternaria* spp. [15] and the bacterial BCA, *P. agglomerans* [16]. This method was used effectively to monitor viable populations of *P. agglomerans* in the phyllosphere of citrus fruits [17].

The objective of this study was to develop and optimise a method with sufficient competence for the quantification of viable populations of *Bacillus subtilis* QST 713 and *Gliocladoum catenulatum* J1446 in commercial formulations. The method for these two BCAs using the PMA approach examined whether PMAxx™ (Biotum, Hayward, CA, USA) could be applied to inhibit DNA amplification of dead cells in the formulated products. The use of the PMA-based qPCR quantification of viable BCA populations was then validated in studies on the temporal changes in the viable inoculum in the phyllosphere of lettuce leaves.

## 2. Materials and Methods

### 2.1. The Biocontrol Agents Used: B. subtilis QST 713 and G. catenulatum J1446

Serenade ASO (Fargro, Arundel, U.K.), a biocontrol product containing *B. subtilis* strain QST 713, was purchased as a liquid formulation from Fargro, UK. Serenade ASO was stored at room temperature. PreStop, a fungal biocontrol product containing *G. catenulatum* strain J1446, was also purchased from Fargro as a wettable powder formulation. Both these formulated BCAs were sterile as plating of the formulations on Nutrient Agar (NA, (Thermo Fisher Scientific Oxoid Ltd, Basingstoke, Hampshire, U.K.) or Malt Extract Agar (MEA, Thermo Fisher Scientific Oxoid Ltd, Basingstoke, Hampshire, U.K.) culture media showed only the growth of the specific BCA. PreStop was stored in a cool dry place at <8 °C, and once opened was frozen at −20 °C. The batch of both the biological products was less than six-months-old when used in experiments.

### 2.2. Cell Suspensions and Viability of Biocontrol Population Treatments

For both biocontrol products, viable populations were determined by quantifying colony-forming units (CFUs) using the viable plate count technique. The total populations were estimated with a Neubauer haemocytometer under a microscope. Cell viability was confirmed by culturing *B. subtilis* on NA and *G. catenulatum* on MEA at 25 °C for 48 h. The non-viable population was estimated as the difference between the total counts made with the haemocytometer and the cultured viable populations. The two products were serially diluted with the maximum recovery diluent (Merck Life Science UK Limited, Gillingham, Dorset, U.K.). For statistical analyses all the data for CFUs was log transformed. After dilution, 500 μL of the BCA suspension was transferred into a 1.5 mL Eppendorf, the total cell concentration in the 500 μL volume was used. This showed that in this volume the *B. subtilis* cells/propagules present was 7.96 log_10_ CFUs, and of these 6.96 log_10_ CFUs were viable. The total conidial concentration in the 500 μL volume of the *G. catenulatum* contained 8.64 log_10_ units, of which 5.7 log_10_ CFUs were viable in the formulation.

### 2.3. PMAxx™ Treatment

PMAxx™ was diluted in DEPC water to produce a 2.5 mM concentration and stored at −20 °C. The optimised PMAxx™ treatment was found to be 25 μM for *B. subtilis* and 50 μM for *G. catenulatum*. This was followed by an incubation stage in which BCA suspensions were encased in aluminium foil and placed into a lightproof sealable container, which was shaken on a rocker at 35 rpm for 15 min. In a cold room (4–8 °C) a photo-activation system was assembled with a rocker fitted with an icebox that contained ca. 2 L ice, with the interior covered by two layers of aluminium foil (reflective side), and two light sources with a light output of 800 W fitted on the top at an angle of ca. 45°. After incubation, cell suspensions were transferred into the system at a distance of 20 cm from the light source to initiate photo-activation. Photo-activation consisted of 1 min light treatment followed by 2 min cooling for 30 cycles with constant agitation at 35 revs/min [3]. After photo-induced cross-linking of PMAxx™ and exposed DNA in dead cells, BCA suspensions were centrifuged at 5000× *g* for 10 min at 4 °C, and the supernatant discarded.

### 2.4. DNA Extraction and qPCR

Genomic DNA from cell pellets was extracted with TRI Reagent^®^ (Sigma–Aldrich) following the manufacturer’s protocol. Extracted DNA was filtered with Millex-VV syringe filter unit 0.1 µm (PVDF, 33 mm and gamma sterilized) and the purity and concentration determined on a NanoDrop spectrometer (NanoDrop ND-1000; NanoDrop Technologies, Wilmington, Delaware, U.S.A.). The integrity of the DNA was determined by electrophoresis on a 1.5% agarose gel run at 60 V for 90 min within TAE buffer solution and stained with GelRed (Biotum, Hayward, California, U.S.A.). The DNA was quantified with the ABI-7500 qPCR detection system (Applied Biosystem Division, Perkin-Elmer Co., Foster City, CA, USA). Reactions were prepared in a clear 96 well qPCR plate and sealed with an adhesive cover. The final volume in each reaction for *B. subtilis* was 12 μL, and for *G. catenulatum* was 15 μL. The final volume contained 5 μL SensiFAST™ SYBR^®^ No-ROX Kit (Bioline Meridian Bioscience, London, U.K.), and 35 ng of DNA extracted with and without PMAxx™. *B. subtilis* reactions contained 434 nM of Bs_dnaK1154 forward primer (5′-ACACGACGATCCCAACAAGC-3′), and 434 nM of Bs_dnaK1254 reverse primer (5′-AGACATTGGGCGCTCACCT-3′) [18]. *G. catenulatum* reactions contained 344 nM of Gc1-1 forward primer (5′-CCGTCTCTTATCGAGCCAAGAT-3′), and 344 nM of Gc3-2a reverse primer (5′-GCCCATTCAAAGCGAGGCATTA-3′) [19]. The PCR conditions used for both BCAs were 94 °C for 3 min followed by 40 cycles of 15 s at 94 °C, 30 s at 60 °C and 30 s at 72 °C. In all cases, three replicates of each qPCR treatment including the controls without a template (NTC) were used. The controls included 3–5 μL of DEPC water instead of DNA. Software-defined storage (SDS) software version 1.5.1 (Applied Biosystems Europe B.V., Warrington, Cheshire, U.K.) was used to analyse and calculate Ct (or Cq) values automatically. All samples that reached fluorescence values above the threshold were treated as a positive reaction, which was determined by the software.

### 2.5. Standard Curves of Cycle Threshold to Copy Number of DNA and Viable Population

**Figure 1:**
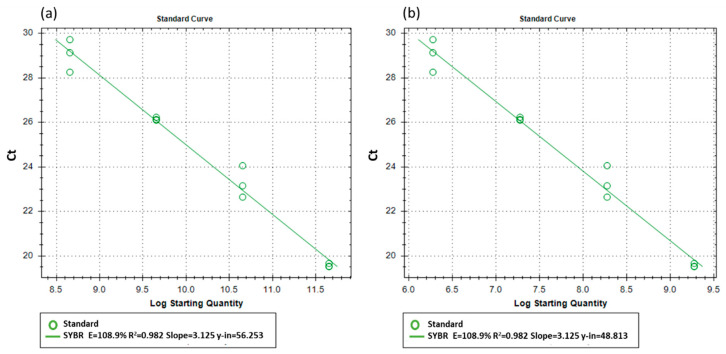
(**a**) The propidium monoazide (PMA)-treated standard curve for *B. subtilis* QST 713 of Ct to copy number generated from genomic DNA; (**b**) The PMA-treated standard curve for *B. subtilis* QST 713 of Ct to log_10_ total number of viable populations.

PMAxx™-treated standard curves were developed for *B. subtilis* and *G. catenulatum*. For *B. subtilis* the DNA from the pure isolate, and for *G. catenulatum* the DNA from the formulated version of PreStop was used to produce standards of Ct to copy number generated (Figure 1a), and Ct to log_10_ total viable populations (Figure 1b). The BCA cell concentrations for standards were PMAxx™-treated. For *B. subtilis* 25 μM, while for *G. catenulatum* 50 μM PMAxx™ concentration was used to treat standard cell suspensions. For both BCAs, for each standard concentration haemocytometer counts were used to determine the total cell concentration, and viable plate count technique used for the total viable cell concentration.

We used the ΔCt values [20] to estimate the number of dead cell populations, which was possible without the use of a standard curve since the following equation could be applied:

ΔCt = Ct value obtained without PMAxx™ (Negative control)—Ct value obtained with PMAxx™ (Treatment).

However, to estimate the number of viable or dead cells, we need to produce a standard curve of Ct values relating to viable populations.

To generate such a standard curve, the following steps were made: (1) BCA cell concentrations with known viable populations were serially diluted using 10 fold dilutions, (2) following this, these standards were PMAxx™-treated, (3) viable cell concentrations were re-determined with the viable plate count technique for each dilution series of the standard points, and (4) subsequently DNA extraction from each dilution series containing a specific BCA standard cell concentration and this produced a linear relationship between Ct value and the number of viable BCA cells. Thus, the standard curve was actually based on the concentration of viable BCA cells extracted instead of DNA copy number produced.

The DNA copy number was used to estimate the viable populations. This required the identification of the linear relationship between the DNA copy number numbers present (Ct) and the CFUs this was extracted from. This information was used to develop the standard curve using three replicates per treatment for the four standard concentrations. Figure 1a shows the relationship between the copy number of the DNA marker (log_10_ 11 to 8 units). For obtaining the total viable populations in a sample, the Ct value was used to estimate the DNA marker copy number through the use of the standard curve. With the use of efficient primers [18,19], there was a linear relationship between the copy number of the target DNA sequence and the Ct values (see Figure 1b).

The same standard curve production procedure of Ct to copy number and log_10_ viable population was carried out for *G. catenulatum* J1446.

### 2.6. Optimisation of the PMAxx-PCR Assays

Toxicity assays: The toxicity effect of PMAxx™ concentrations was tested with log_10_ viable populations of cells at 4, 5, 6, 7, 8 and 9 log_10_ CFUs/mL for *B. subtilis*, and 2, 3, 4, 5, 6, and 7 log_10_ CFUs/mL for *G. catenulatum*. Each tested concentration contained one independent biological replicate, and this was plated a total of four times to produce four technical replicates in each of the six cell concentrations for each of the four PMAxx™ concentration treatments (12.5 μM, 25 μM, 50 μM and 100 μM). Negative control (0 μM PMAxx™) treatments were included for each cell concentration. Thus, there were thirty treatments (six cell concentrations each tested at five PMA contractions), each with four technical replicates.

The effect of PMAxx™ on cell viability was analysed with Analysis of Variance (ANOVA). The data on BCA population size were log_10_ transformed prior to the ANOVA. All statistical significances were judged at a *p* value of 0.05; GenStat (version.18) statistical package was used.

Heat treatment for assays of ratios of viable/dead cells: To increase the total number of dead cells, the cell suspensions of 500 μL containing the BCAs were treated with heat on a dry-block heater. The first heat treatment was at 95 °C for 5 min, and this increased the total number of dead cells from 1 log_10_ units to 2.75 log_10_ units for *B. subtilis* (as estimated by plating). The same heat treatment for *G. catenulatum* increased the total number of non-viable propagules from 2.94 to 5.75 log_10_ units. The second heat treatment was at 95 °C for 10 min. This increased the total number of dead cells from 1 log_10_ unit to 3.44 log_10_ units of dead cells for *B. subtilis*. The second heat treatment for *G. catenulatum* increased the total non-viable propagules from 2.94 to 6.22 log_10_ units.

However, it should be noted that the commercial formulated versions of the BCAs contained a large proportion of dead cells. As a result of this, a separate experiment was set up in which the serial dilution method was used to dilute the number of dead cells present in the BCA suspensions, and for *B. subtilis* this was carried out with the pure isolate. For *G. catenulatum* this was carried out with the formulated version. The total cell concentration and the total number of dead cells were as follows: for *B. subtilis* 1 log_10_ unit of cells were already dead, and for *G. catenulatum* 3.21 log_10_ units of cells were dead. These BCA suspensions were serially diluted, and from each serial dilution, this changed the total number of dead cells by a factor of 10, i.e., for *B. subtilis* this changed the dead cells to 0.1 log_10_ units, while for *G. catenulatum* this changed the total dead cells to 2.21 log_10_ units. The BCA solutions were further serially diluted four times to decrease the number of dead cells by up to a factor of 10,000. The exact mean total numbers of dead cells and the total number of cells are provided in Supplementary Table S1.

Statistical analyses of the blocking of DNA quantification of dead cells with PMAxx™ was done in two experiments for each BCA. Each experiment was set-up as a factorial design. The experiments in total contained 8 (*B. subtilis*) and 6 (*G. catenulatum*) biological replicates for each treatment combination: type of treatment (room, heat treatment of 95 °C for 5 min, and 95 °C for 10 min) and PMAxx™ concentration (0 μM, 12.5 μM, 25 μM, 50 μM and 100 μM). We used ΔCt values to estimate the number of dead cells. The effect of PMAxx™ in suppressing DNA amplification of dead cells in relation to heat treatment, and the sensitivity of this quantification method were assessed through a restricted maximum likelihood (REML) analysis. In the REML analysis, the fixed factor was the PMAxx™ concentrations. The Ct data were used for the REML analysis and therefore were not transformed. The serial dilution treatments were pooled from two standard curves for the PMAxx™ and non-PMAxx™-treated. These treatments were not included in statistical analysis and were solely used for graphical purposes as a representative of ΔCt (signal reduction) for BCA suspensions with concentrations that contained a lower number of dead cells than the room temperature treatments.

### 2.7. Testing for Viable BCA Quantification with the Optimised PMAxxTM-qPCR from the Phyllosphere of Lettuce

For these studies *B. subtilis* QST 713 colonies were cultured on nutrient agar, divided into four equal parts and transferred to a 1 L vacuum filter flask containing pre-autoclaved Tryptone Soya broth (Sigma) and grown on a rotary shaker (110 rpm) at 20–25 °C for 10 days. For *G. catenulatum* J1446 inoculum, 5 g of PreStop powder was placed in 1 L of tap water and hand shaken vigorously prior to use.

The formulated Serenade ASO product of *B. subtilis* QST 713 was not directly used because the formulation components had qPCR inhibiting materials and additives that affected the standard curve for estimating the viable populations. Before spraying, total populations were estimated with a Neubauer haemocytometer, and the total viable cells by serial dilution.

BCA dynamics were studied in two environments: polytunnel and glasshouse. At each of the six sampling time points, the oldest leaves of five independent lettuce plants (the single oldest leaf from each plant) were sampled and pooled together as a composite sample to estimate viable populations of *B. subtilis* QST 713 (pure isolate) and *G. catenulatum* J1446 (formulated) in the phyllosphere of the lettuce leaves.

All experiments followed seven common steps:

(1) Plant propagation and selection; plants (*Lactuca sativa* cv. Carter) were sown, grown, and selected for being pest and disease-free and healthy, with a minimum of six leaves.
(2) BCA cultivation and concentration calibration; plate counts were used to determine the concentration of the cultivated BCAs and were adjusted as necessary to obtain 8 log_10_ CFUs/mL for *B. subtilis*, and 8 log_10_ spores/mL for *G. catenulatum*.
(3) Plant treatment; plants were sprayed with the appropriate BCA as a fine droplet setting until just before run-off.
(4) Plant drying; after treatment plants were allowed to dry for 1 h in the glasshouse, and then placed into their designated glasshouse and/or polytunnel.
(5) Sampling was done on days 0 (1 h after BCA application), 2, 4, 6, 8 and 10 after spraying. On each sampling day the oldest leaf was collected from five pre-determined plants (one leaf per plant), and immediately pooled and placed into a falcon tube containing maximum recovery diluent (Sigma).
(6) Surface washing, filtration, and cell pellet collection; the leaves were soaked in maximum recovery diluent until full, sealed, and shaken on a rotary shaker at 100 rpm for 30 min at 10 °C. The contents were filtered with a wet muslin cloth (four layers), and the cells were pelleted by centrifugation at 2000× *g* for 15 min at 4 °C. The supernatant was decanted, and the cell pellet supplemented up to 500 μL with maximum recovery diluent solution, and then transferred into a 1.5 mL Eppendorf tube for storage at 4 °C.
(7) PMAxx™ treatment (25 μM for *B. subtilis*; 50 μM for *G. catenulatum*) and the DNA extraction and qPCR methods were previously described.

## 3. Results

### 3.1. Effect of PMAxx™ on Blocking DNA Amplification of Dead BCA Cells

**Figure 2:**
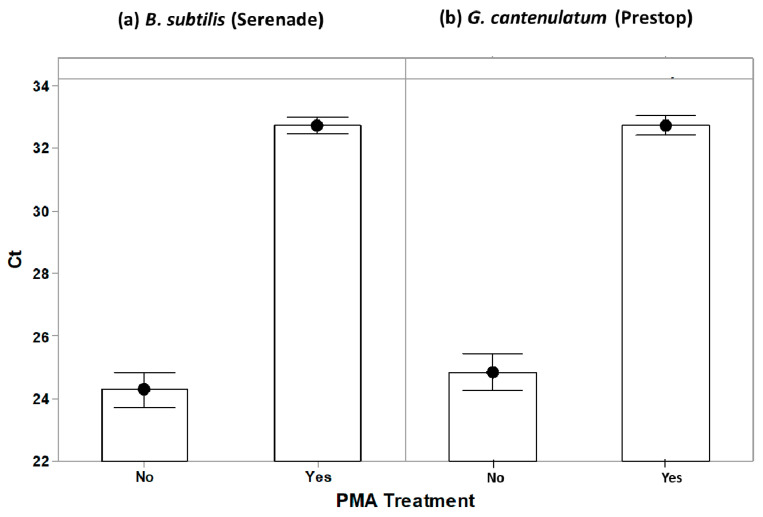
Effect of PMAxx™ on blocking DNA amplification from dead BCA cells for (**a**) *B. subtilis* and (**b**) *G. cantenulatum*. The black circular points show the mean of six replicates for the non-PMA treatment, and the mean of 24 replicates for the PMA treatment. Each replicate was carried out in triplicate on the qPCR plate. The bars represent the standard error of the means.

**Figure 3:**
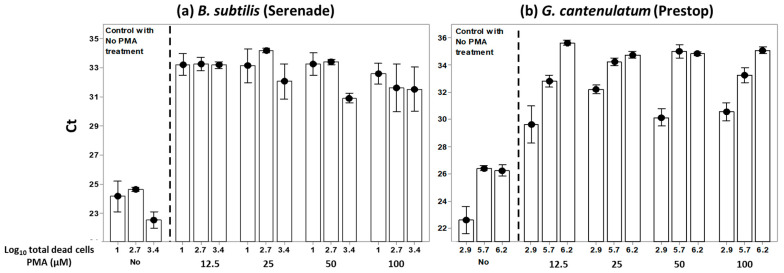
(**a**) Impact of PMAxx™ concentrations on blocking DNA amplification of increasing log_10_ total dead cells from *B. subtilis*; (**b**) Impact of PMAxx™ concentrations on blocking DNA amplification of increasing log_10_ total dead cells from *G. catenulatum*. Each treatment combination contained two independent biological replicates, and each biological replicate was carried out in triplicate on the qPCR plate. The mean is represented by the black circles, and the bars represent the standard error of the means.

This experiment was set-up for testing the effect of PMAxx™ on blocking DNA amplification from dead BCA cells. Cell suspensions of *B. subtilis* and *G. catenulatum* with three different total numbers of dead cells were subjected to PMAxx™ dosages of 0 (control), 12.5, 25, 50, and 100 μM. For *B. subtilis* the numbers of dead cells were 1, 2.75, and 3.44 log_10_ units, while for *G. catenulatum* these were 2.94, 5.70 and 6.22 log_10_ units (see Supplementary Table S1). The heat treatment method was used to increase the number of dead cells. PMAxx™ blocked the amplification of the DNA from the dead BCA cells for both species (*p* < 0.01; Figure 2). This blocked amplification of increasing numbers of dead cells in each tested PMAxx™ concentration (*p* < 0.05), as shown in Figure 3a,b. For both BCAs, the mean Ct from the PMAxx™ treatment varied with the number of dead cells present (*p* < 0.05; see Supplementary Tables S2 and S3 for the summary of the statistical analyses).

### 3.2. PMAxx™ Concentration Response at Different Numbers of Total log_10_ Dead Cells

**Figure 4:**
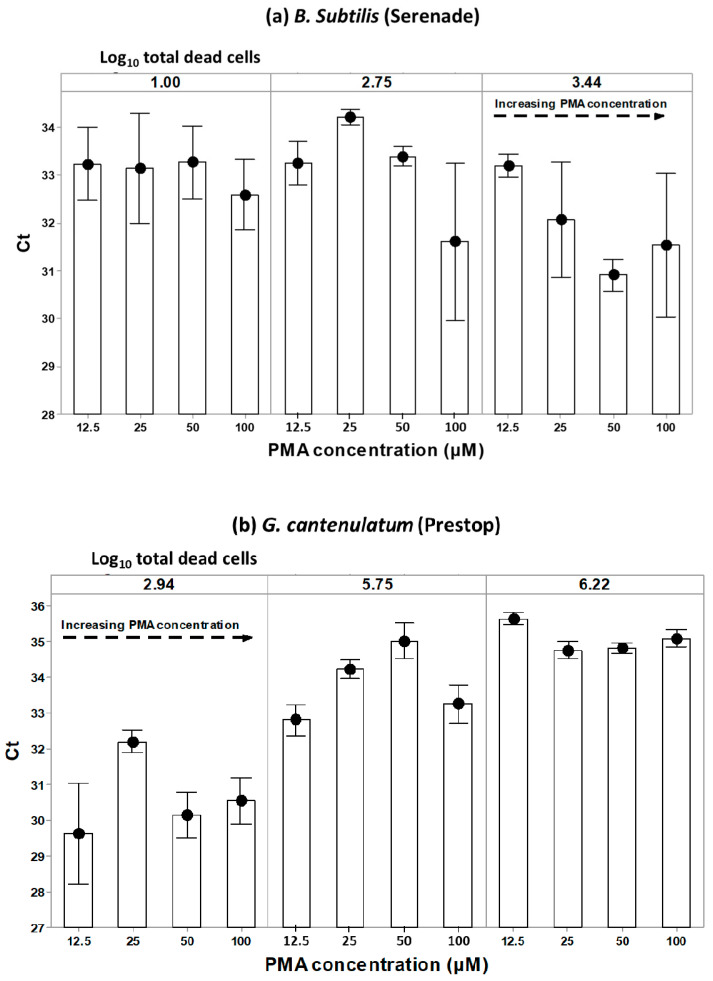
(**a**). Dose response of cycle threshold (Ct) to increasing PMAxx™ concentrations on the same total numbers of *B. subtilis* dead cells; (**b**) Dose response of cycle threshold (Ct) to increasing PMAxx™ concentrations on the same total numbers of *G. catenulatum* dead cells. The points show the mean of two independent biological experiments carried out with three replicates for the qPCR plate. The bars represent the standard error. The detection limit was Ct < 35.

The data collected for investigating the effect of PMAxx™ on blocking DNA amplification from dead cells were examined in relation to the increase in logarithmic numbers of dead cells and the parallel increase in the PMAxx™ concentration (Figure 4). This enabled the examination of the impact of increasing PMAxx™ concentration on Ct values for the same number of log_10_ total dead cells. For *B. subtilis* the total number of dead cells tested were 1, 2.75 and 3.44 log_10_ units, while for *G. catenulatum* this was 2.94, 5.75 and 6.22 log_10_ units, respectively. Each cell suspension was subjected to PMAxx™ concentrations of 12.5 μM, 25 μM, 50 μM, and 100 μM. Increasing PMAxx™ concentration had no effect on the Ct value of the same concentration with the total dead cell number. Figure 4a,b show the mean Ct values vs. PMAxx™ concentrations for the different concentrations of the dead cells. However, there were no significant (*p* > 0.05) differences in Ct values when PMAxx™ concentrations were increased in each of the tested total dead cell concentrations examined.

### 3.3. Assays for Testing PMAxx™ Concentration on In Vitro Viability Counts of the Two BCAs

**Figure 5:**
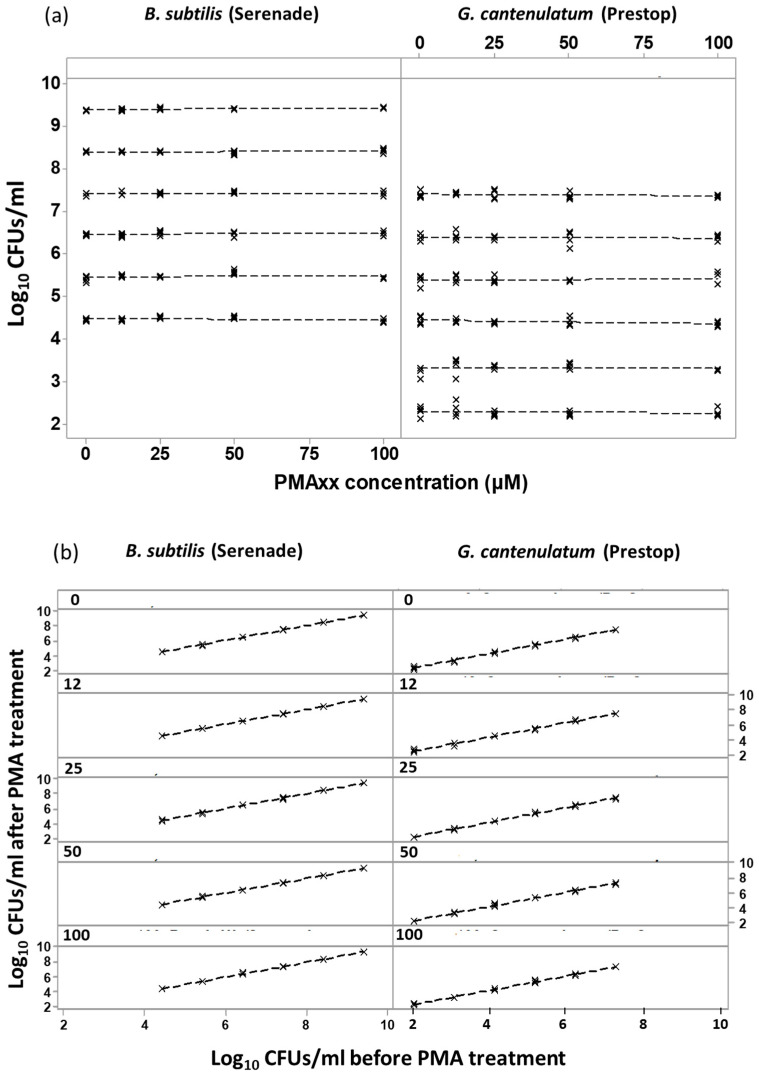
(**a**) Effect of increasing PMAxx™ dose on viable plate counts; (**b**) Linear standard curves of the relationship between CFUs mL^−1^ before and after PMAxx™ treatment. The PMAxx™ concentration response with R2 values for *B. subtilis* are between 0–36%, while for *G. catenulatum* ranged between 0–16%. Each biological replicate was plated four times to confirm CFUs mL^−1^, and therefore each treatment combination contains four technical replicates. The standard error for each treatment was <1.25.

This experiment was set-up for testing the effect of PMAxx™ concentration on viable CFU counts on agar plates. Log_10_ CFUs mL^−1^ of 4, 5, 6, 7, 8, and 9 for *B. subtilis* and 2, 3, 4, 5, 6, and 7 for *G. catenulatum*, respectively were treated with 0 μM (negative control), 12.5 μM, 25 μM, 50 μM, and 100 μM of PMAxx™ and then plated. For both BCAs in all tested BCA concentrations, PMAxx™ doses had no impact on the CFU/mL (*p* > 0.05) (Figure 5). This data set was also used to determine the relationship between the tested PMAxx™ concentrations on a range of CFUs mL^−1^. Each tested PMAxx™ concentration and the negative associated control were plotted as a linear standard curve to show the PMAxx™ impact, if any, on producing standard curves with PMAxx™-treated samples. The relationship between CFUs mL^−1^ before and after treatment with PMAxx™ is shown in Figure 5a. All fitted linear standard curves had R^2^ values of >99.6% (Figure 5b). Supplementary Tables S4 and S5 provide the summary statistical analyses for these assays.

### 3.4. Sensitivity Range of PMAxxTM-qPCR on Blocking DNA from Dead Cells

The data collected for investigating the effect of PMAxx™ on blocking DNA amplification from dead cells were pooled and arranged in relation to log_10_ total number of dead cells. The ΔCt from each PMAxx™ treatment concentration was pooled together because (1) all concentrations with PMAxx™ blocked DNA amplification from all the tested numbers of dead cells in the qPCR stage significantly (*p* < 0.05); (2) increasing the concentration of PMAxx™ from 12.5 μM had no significant effect on the ΔCt values at the same total dead cell concentration (*p* > 0.05); and (3) the tested PMAxx™ concentrations did not reduce the amount of CFUs/mL (*p* > 0.05). For *B. subtilis* the total number of dead cells plotted in log_10_ units were 0.0001, 0.001, 0.01, 0.1 (serial dilution treatments), 1 (room temperature), 2.75 and 3.44 (heat treatments), while for *G. catenulatum* the total number of dead cells in log_10_ units plotted were 0.021, 0.21, 1.21 (serial dilution treatments), 2.94 (room temperature), 5.75 and 6.22 (heat treatments).

**Figure 6:**
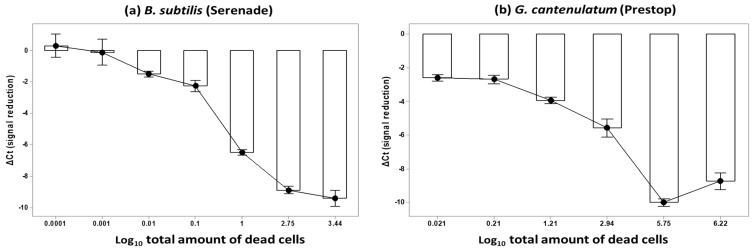
(**a**) Interval plot of overall mean ΔCt (signal reduction) to overall mean log_10_ total dead cell number of *B. subtilis;* (**b**). The interval plot of overall mean ΔCt (signal reduction) to overall mean log_10_ total dead cell number of *G. catenulatum.* Log_10_ mean numbers of total dead cells for all treatments were determined from the difference between total cell counts using a haemocytometer and the viable plate counts. The bars represent the standard error of the means.

For both BCAs the increase in the number of log_10_ total dead cells led to the increase in the ΔCt (signal reduction *p* < 0.05; Figure 6a,b). The viable population reduction method of heat treatment had an independent effect of decreasing Ct in *B. subtilis* and increasing Ct of *G. catenulatum*, and this affected the ΔCt values (*p* < 0.05). In both these figures, the relationship of ΔCt (signal reduction) values to the mean log_10_ number of total dead cells for *B. subtilis* showed the blocking ability for the PMAxx™-qPCR tool of at least up to 3.44 log_10_ CFUs. For *G. catenulatum* this was blocked by the PMAxx™-qPCR at up to 5.75 log_10_ cells. See Supplementary Tables S6 and S7 for summary statistical analyses of the data sets.

### 3.5. Temporal Fluxes in Phyllosphere Quantification of the Two BCAs on Lettuce Leaves Using the PMAxxTM-qPCR Technique

**Figure 7:**
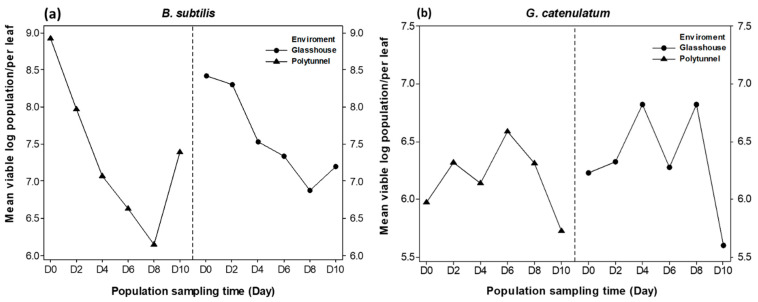
(**a**) Temporal viable population of *B. subtilis* in glasshouse and polytunnel grown lettuce; (**b**) the temporal viable population of *G. catenulatum* in glasshouse and polytunnel grown lettuce. The standard errors were <1.25.

The PMAxx™-qPCR technique for both BCAs was tested in practice by carrying out experiments on lettuce leaves of crops grown in two semi-commercial environments (polytunnels and glasshouse). Viable population of *B. subtilis* declined in both tested environments, yet the pattern of decline was more rapid in the polytunnel than in the glasshouse (Figure 7a). However, there was some spike in viable populations in the phyllospere of the lettuce leaves on day 10. For *G. catenulatum*, there were much smaller temporal viable populations with similar fluxes in populations observed in both the glasshouse and polytunnel systems (Figure 7b). It should be noted that it was difficult to produce standard curves for Serenade ASO with the serial dilution method and the pure isolate *B. subtilis* QST 713 was used in these studies.

## 4. Discussion

The PMAxx™-qPCR approach provided accurate tools for the monitoring of the viable BCA populations because of its efficient blocking ability of the DNA of the dead cells of these two BCAs. This allowed the accurate quantification of viable but non-culturable cells. This study focused on selection of the PMAxx™ dose necessary to minimise inhibitory effects of PMAxx™ on amplification of DNA from viable BCAs in their commercial formulation and tested the limits of this method in blocking the DNA of the dead cells in the formulations. In addition, it was shown that this PMAxx™-qPCR can track the viable populations of these two BCAs in the phyllosphere of lettuce plants in two semi-commercial production systems. It also provides information on the population dynamics in the complex environment of the phyllosphere. The observations on the fluctuations in the viable propagules of these BCAs in the two culture systems assayed informs on both the activation times of the *B. subtilis* spores or the conidiogenesis cycles in the case of *G. catenulatum.* This provides benefits in obtaining critical information on the effect of interacting abiotic factors, host development stages, and agronomic practices on the fate of such BCAs in the phyllosphere of economically important horticultural crops [21].

Previously, more traditional methods (culture-dependent) and independent techniques [2,22], quantitative PCR for bacteria [7], yeasts [9], and for fungal BCAs [23] have been developed. However, these do not usually separate dead and viable cells/propagules or non-culturables. The advantage of the PMA-qPCR technique is that it can be utilised to monitor viable population of BCAs. For example, *P. agglomerans* CPA-2 [16] and *Lactobacillus plantarum* PM411 [24] have been successfully monitored on aerial plant surfaces. The PMAxx™-qPCR approach was recently used for distinguishing dead cells from live ones within microbial ecosystems [25].

For the development of this tool for tracking these two commercial BCAs the most critical aspect was the selection of the right PMAxx™ dose. Optimal PMAxx™ doses were selected based on several criteria, including blockage of amplification of dead cells, preservation of live cells, and reliable signal reduction. The tested dosages of 12.5–100 μM showed no toxic effects on population densities of *B. subtilis* (log_10_ 4 to 9 CFUs mL^−1^) and *G. catenulatum* (log_10_ 3 to 7 CFUs mL^−1^). Previously, no toxicity was found with 50 μM dosage for detection of *P. agglomerans* CPA-2 [17], whereas significant cytotoxic effects on *L. monocytogenes* were detected with the same concentration [26]. A study on *Legionella pneumophila* suggested minimal toxicity effects occurred at 100 μM, whereas a 200 μM treatment was toxic and reduced viable cells by 0.4 log_10_ units [27].

In the present study, the lowest dose of PMAxx™ caused a plateau for both the bacterial and fungal BCAs in all three different cell suspensions with different numbers of total dead cells. This suggests that exploration of low PMAxx™ concentrations can be critical to identify the dose response relationship of dead cell densities to PMAxx™. However, studies on *Legionella* biofilms identified no significant PMA (30 μM–100 μM) effects of blocking DNA from dead cells [28].

An important parameter to consider is the matrix of the cells when dealing with formulated BCAs, due to the impact of the components on DNA amplification efficiency and PMA uptake [29]. Formulated BCAs were amplifiable when filtration was used after DNA extraction. However, obtaining appropriate standard Ct curves to quantify the true populations instead of relative values was difficult as the concentration of formulation additives (dyes, adjuvants) and their relative effect on the qPCR process were unknown. In addition, it is difficult to separate the BCA cells from formulation additives because some of these dyed the DNA of the organism. The proprietary knowledge of the actual formulations meant that we were unable to obtain detailed information of the additives and adjuvants actually used in the commercial formulations of these two BCAs. Despite this, there was a close correlation in estimated viable population sizes between the PMAxx™-qPCR and viable plate count technique when tested in laboratory environments. Previous studies had developed DNA markers for each of these BCAs for monitoring populations in vivo in some different environments [18,19]. Studies on *E. coli, Staphylococcus aureus,* and *L. monocytogenes* used similar population densities of dead cell concentrations as we have used, including minimising pre-conditioning of cells to avoid impacts on the quantified viable population [30]. The present study has overall shown that 25 μM for *B. subtilis* and 50 μM for *G. catenulatum* are the optimal PMAxx™ concentrations that can satisfy the criteria of blocking amplification of dead cells, preserving live cells, reliable signal reduction, and overcoming possible formulation matrix inhibitory effects. Some previous studies with bacterial pathogens such as *E. coli* and *Salmonella* have found similar ranges [14,31].

Some of the differences observed between the PMAxx™-qPCR and viable plate counts could be due to the presence of viable but non-culturable cells with compromised membranes. The initial signal reductions from non-heat-treated cell suspensions were confirmed by microscopic counts for both BCAs, as reported in previous studies [32,33]. Signal reduction with presumed viable cell suspensions of both BCAs is possibly due to membrane compromised cells, particularly for *G. catenulatum* J1446. This is because the freeze-drying process can cause distress to microorganisms, possibly modifying the permeability of the cytoplasmic membrane and damaging the cell wall [34,35,36]. Liquid formulations also involve a form of cell drying and can contain dyes, additivities, and adjuvants; the effects of these additional factors on cell mortality, cytoplasmic membrane integrity, and cell wall properties could explain the initial signal reduction observed for *B. subtilis* QST 713 [37]. PMAxx™ may bind DNA of damaged cells, but these injured cells may repair their membrane and persist as viable but not culturables [38].

Further studies with the non-formulated strain of *B. subtilis* QST 713 were more promising and hence used for further studies in the present research to confirm that the PMAxx™-qPCR system blocked the DNA of dead *B. subtilis* cells in the lettuce phyllosphere. In contrast, the use of the non-formulated strain of *G. catenulatum* J1446 proved difficult for the quantitative DNA extraction. However, this might partially be due to the cultivation of this fungal BCA on solid agar media instead of in liquid culture. Thus, the formulated *G. catenulatum* BCA was used in the confirmation study of PMAxx™-qPCR on blocking DNA from dead *G. catenulatum* cells in the lettuce phyllosphere. Preliminary studies with *G. catenulatum* suggested that the selected formulation type (dry) and possibly the choice of dye (white) were beneficial to the PMAxx™-qPCR process in comparison to using the pure isolate [21]. In the present study, a mean difference of 10 cycles representing 3.44 log_10_ reductions for *B. subtilis* and 5.75 log_10_ reductions for *G. catenulatum* were found after heat treatment followed by a PMAxx™ treatment. The heat treatment method employed to increase the numbe of dead cells may have also changed the amount of DNA readily extractable and quantifiable and, therefore, the Ct values [39]. As a result of this, the number of dead cells tested could have produced a lower signal reduction at the largest relative values of dead cells, as they were subjected to a longer period of thermal heat treatment. Therefore, there is a rationale for using UV light treatment in reducing viable populations in future studies. Furthermore, if necessary, the excess number of pre-present dead cells in formulated versions can be overcome with addition of excess viable cells from an independently cultivated source.

Mean viable population mortality to mean signal reduction (non-PMAxx™ -PMAxx™-treated) increased on the log_10_ scale at least up to the value of 3.44 units for *B. subtilis,* and up to the 5.75 units for *G. catenulatum*. The mean signal reduction was related to the numbers of dead cells in the interval plots for representing the limit of the PMAxx™ blocking ability. The limit of dead cell quantification inhibition has previously been reported for probiotic bacteria [40], *L. plantarum* PM411 [26] and *P. agglomerans* CPA-2 [16] but with varying ranges.

The studies on temporal changes in viable populations of both BCAs in the phyllopshere of lettuce leaves were carried out for the first time. This showed that overall, there was a decrease in the populations of both BCAs isolated from lettuce leaves using the developed tools but with clear differing patterns. This could have implications for the level of control achievable as such BCAs require a relatively high concentration of viable populations for efficacy. The temporal decrease in populations may thus influence the levels of control of fungal pathogens achieved. Previous studies of *P. agglomerans* CPA-2 had a maximum of 16 cycle difference representing 10^5^ CFUs/mL [16]. The development of such approaches to quantify viable populations of BCAs is critical if they are to provide consistent threshold levels for effective biocontrol. In addition, the resilience of such BCAs under future climate-related scenarios may require platform monitoring systems for the designing of better formulations for improved BCA survival, and development of BCA application strategies to improve disease control efficacy.

## 5. Conclusions

This study developed the molecular tool PMAxx™-qPCR for quantifying viable population in commercial formulations, and in environmental samples for *B. subtilis* QST 713 and *G. catenulatum* J1446. The method is a valuable tool for studying the kinetics and ecological fate of BCAs under natural environmental conditions to optimise their use for disease management.

## Data Availability

All the raw data for these studies are available via NIAB-EMR where they are archived. These are available via Prof. X.X.

## Supplementary Materials

The following are available online at https://www.mdpi.com/2079-7737/10/3/224/s1, Table S1. List of treatments to produce different BCA cell concentrations. Note: Serial dilution series 1 for *G. catenulatum* was not included because the PMA treated counterpart was unidentifiable. Table S2. The Restricted Maximum Likelihood (REML) variance components analysis of PMAxx™ dosages on DNA amplification for *B. subtilis*/Serenade. Table S3. The REML variance components analysis of PMAxx™ dosages on DNA amplification for *G. catenulatum*/PreStop. Table S4. The REML variance components analysis in effect of PMAxx™ concentration on BCA CFUs for *B. subtilis*/Serenade. Table S5. The REML variance components analysis in effect of PMAxx™ concentration on BCA CFUs for *G. catenulatum*/PreStop. Table S6. The REML variance components analysis of increasing PMAxx™ dose and assay sensitivity for *B. subtilis*/Serenade. Table S7. The REML variance components analysis of increasing PMAxx™ dose and assay sensitivity for *G. catenulatum*/PreStop.
