# Supplementary material for: Molecular Assay Development to Monitor the Kinetics of Viable Populations of Two Biocontrol Agents, Bacillus subtilis QST 713 and Gliocladium catenulatum J1446, in the Phyllosphere of Lettuce Leaves
Source: Biology (Basel). 2021 Mar 15;10(3):224. doi: 10.3390/biology10030224 (PMC8001495; doi:10.3390/biology10030224)
Supplement: Supplementary file 1 [file biology-10-00224-s001.pdf]

Suppl. Table 1. List of treatments to produce different BCA cell concentrations. Note: Serial dilution series 1 for *G. catenulatum* was not included because the PMA treated counterpart was unidentifiable.

Treatment	Organism	Formulated	Total volume (µL)	Mean total cell concentration (log_{10}) confirmed with Haemocytometer	Mean total amount of live cells (log_{10}) confirmed with plate counts	Mean total amount of dead cells (log_{10})
Room temperature	*B. subtilis*	Yes	500	7.96	6.96	1.00
Room temperature	*G.catenulatum*	Yes	500	8.64	5.7	2.94
95 °C for 5 mins	*B. subtilis*	Yes	500	7.96	5.21	2.75
95 °C for 5 mins	*G.catenulatum*	Yes	500	8.64	2.89	5.75
95 °C for 10 mins	*B. subtilis*	Yes	500	7.96	4.52	3.44
95 °C for 10 mins	*G.catenulatum*	Yes	500	8.64	2.42	6.22
Serial dilution 1	*B. subtilis*	No	500	9.40	9.3	0.10
Serial dilution 2	*B. subtilis*	No	500	8.40	8.39	0.01
Serial dilution 2	*G.catenulatum*	Yes	500	6.60	5.39	1.21
Serial dilution 3	*B. subtilis*	No	500	7.40	7.40	0.00
Serial dilution 3	*G.catenulatum*	Yes	500	5.60	5.39	0.21
Serial dilution 4	*B. subtilis*	No	500	6.40	6.40	0.00
Serial dilution 4	*G.catenulatum*	Yes	500	4.60	4.58	0.02

Suppl. Table 2. The Restricted Maximum Likelihood (REML) variance components analysis of PMAxx™ dosages on DNA amplification for *B. subtilis*/Serenade

REML variance components analysis

Tests for fixed effects

Sequentially adding terms to fixed model

Fixed term	Wald statistic	n.d.f.	F statistic	d.d.f.	P value
PMA_concentration_M	164.93	4	41.23	37.0	<0.001
Condition_treatment	7.97	2	3.98	37.0	0.027
PMA_concentration,_M.Condition_treatment	6.62	8	0.83	37.0	0.584

Suppl. Table 3. The REML variance components analysis of PMAxx™ dosages on DNA amplification for *G. catenulatum*/PreStop

REML variance components analysis

Tests for fixed effects

Sequentially adding terms to fixed model

Fixed term	Wald statistic	n.d.f.	F statistic	d.d.f.	P value
PMA_concentration_M	320.43	4	80.11	75.0	<0.001

Condition_treatment	98.08	2	49.04	75.0	<0.001
PMA_concentration_M.Condition_treatment	10.27	8	1.28	75.0	0.265

Suppl. Table 4. The REML variance components analysis in effect of PMAxx™ concentration on BCA CFUs for *B. subtilis*/Serenade

Analysis of variance

Variate: log_Ratio

Source of variation	d.f.	s.s.	m.s.	v.r.	P value
log_Conc	5	0.105631	0.021126	14.11	<.001
PMAxx_tm_Conc_M	4	0.035140	0.008785	5.87	<.001
log_Conc.PMAxx_tm_Conc_M	20	0.072983	0.003649	2.44	0.002
Residual	90	0.134723	0.001497		
Total	119	0.348477			

Suppl. Table 5. The REML variance components analysis in effect of PMAxx™ concentration on BCA CFUs for *G. catenulatum*/PreStop

Analysis of variance

Variate: log_Ratio

Source of variation	d.f.	s.s.	m.s.	v.r.	P value
log_Conc	5	0.271705	0.054341	5.55	<.001
PMAxx_tm_Conc_M	4	0.064872	0.016218	1.66	0.167
log_Conc.PMAxx_tm_Conc_M	20	0.094571	0.004729	0.48	0.967
Residual	90	0.881428	0.009794		
Total	119	1.312577			

Suppl. Table 6. The REML variance components analysis of increasing PMAxx™ dose and assay sensitivity for *B. subtilis*/*Serenade*

REML variance components analysis

Tests for fixed effects

Sequentially adding terms to fixed model

Fixed term	Wald statistic	n.d.f.	F statistic	d.d.f.	P value
PMA_concentration_M	3.95	3	1.32	33.0	0.286
Condition_treatment	24.17	2	12.08	33.0	<0.001
PMA_concentration_M.Condition_treatment	4.12	6	0.69	33.0	0.662

Suppl. Table 7. The REML variance components analysis of increasing PMAxx™ dose and assay sensitivity for *G. catenulatum*/PreStop

REML variance components analysis

Tests for fixed effects

Sequentially adding terms to fixed model

Fixed term	Wald statistic	n.d.f.	F statistic	d.d.f.	P value
PMA_concentration_M	6.37	3	2.12	58.0	0.107
Condition_treatment	6.69	2	3.35	58.0	0.042
PMA_concentration_M.Condition_treatment	9.08	6	1.51	58.0	0.190
